# Naphthalene Decomposition on Fe(110)—Adsorption, Dehydrogenation, Surface Carbon Formation and the Influence of Coadsorbed Oxygen

**DOI:** 10.1021/acs.jpcc.4c06619

**Published:** 2025-01-27

**Authors:** Lea Hohmann, Franziska Dahlmann, Giorgio Bruno Braghin, Léonie Laviron, Layal Hussein, Jakob Martinez, Anton Harrer, Haley Robertson, Jona Guiborat, Xiaoming Hu, Jonas Weissenrieder, Klas Engvall, Jerry LaRue, Tony Hansson, Mats Göthelid, Amirreza Ghassami, Dan J. Harding, Henrik Öström

**Affiliations:** † Department of Chemical Engineering, KTH Royal Institute of Technology, 100 44 Stockholm, Sweden; ‡ INP-ENSIACET, Université de Toulouse, 31030 Toulouse, France; § Light and Matter Physics, Applied Physics, KTH Royal Institute of Technology, 114 19 Stockholm, Sweden; ∥ Department of Physics, Fysikum, Stockholm University, 106 91 Stockholm, Sweden; ⊥ Wallenberg Initiative Materials Science for Sustainability, Applied Physics, KTH Royal Institute of Technology, 114 19 Stockholm, Sweden; # Schmid College of Science and Technology, Chapman University, Orange, California 92866, United States; ¶ MAX IV Laboratory, Lund University, P.O. Box 118, SE-22100 Lund, Sweden

## Abstract

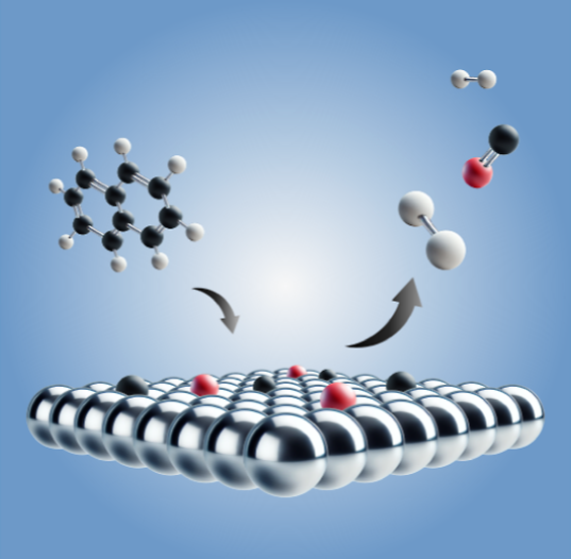

Tar is an undesirable byproduct of biomass gasification, which can be removed through catalytic reforming to syngas components. Iron is a promising, abundant alternative to highly active but toxic nickel catalysts. The results observed so far in catalytic studies with iron have been mixed. In this paper, the decomposition of naphthalene, a representative model compound of tar, was studied on the catalytic Fe(110) surface using temperature-programmed desorption (TPD), sum frequency generation spectroscopy (SFG), and X-ray photoelectron spectroscopy (XPS). Napthalene adsorption, dehydrogenation and the formation of surface carbon were investigated, as well as the influence of oxygen. In comparison with previous studies on Ni(111), a similar dehydrogenation activity was found for Fe(110) with two main H_2_ TPD peaks at 450 and 550 K. The reaction of naphthalene on Fe(110) resulted in the predominant formation of carbidic and atomically adsorbed carbon on the surface, which did not dissolve into the bulk even at high temperatures. A moderately carbon-covered surface was shown to still be active toward naphthalene decomposition. Similarly to Ni(111), large amounts of oxygen inhibited the reaction but, at low oxygen doses, very high hydrogen yields were observed, suggesting that Fe(110) could be a valid alternative for tar decomposition.

## Introduction

Tar, a mixture of polyaromatic hydrocarbons (PAHs), is a byproduct of some industrial processes such as biomass gasification.^1^ Tars are unwanted byproducts which can cause problems such as clogging and fouling,^1^ but they can be catalytically converted in the generic reaction PAH + oxidant → CO + H_2_. For this, nickel steam reforming catalysts are typically used. However, nickel is a toxic material, and it would be desirable to replace it with a more benign and abundant alternative metal.

One suggestion for a possible tar reforming catalyst is iron, the fourth most abundant element in the earth’s crust. Iron has been tested as a catalyst for steam reforming of tars or various model compounds in several catalytic studies.^2−10^ The results are mixed, ranging from no activity to high activity, but in most experiments the performance is below that of Ni catalysts. The poor performance of Fe catalysts is often attributed to its rapid oxidation to various oxide phases, which are thought to have a low catalytic activity.^7,10^ The addition of hydrogen to reduce the catalyst resulted in increased activity in several studies.^2,4^ Another reason sometimes cited for the poor catalytic activity of Fe(110) is the sensitivity to loss of surface area by sintering.^5^

The adsorption and reactions of the simplest aromatic hydrocarbon benzene have been investigated on a range of low-index surfaces of transition metals. Different experimental methods that are sensitive to the surface structure, symmetry, and orientation show that at low temperature and coverage the molecules adsorb with the ring parallel to the surface.^11−13^ The interaction of the π-electron system with the metal reduces the sp^2^ character of the carbons. The partial-sp^3^ hybridization is shown by the C–H bonds moving out of the plane of the ring.^12^ This is the case for benzene on thin Fe(110) films, where it adsorbs in a planar structure with C_3v_ symmetry.^14^ The (110) surfaces of face centered cubic metals Pt, Pd are exceptions, where benzene prefers a tilted adsorption site.^15,16^ At higher temperature, partial dehydrogenation to benzyne (C_6_H_4_) occurs, leading to the formation of metal–carbon bonds and a tilt of the molecules away from the surface.^13,17,18^ Further heating then leads to more dehydrogenation.

There are far fewer studies of the adsorption and reaction of naphthalene. On Pt(111)^19^ and Rh(111)^20^ TPD measurements show similar behavior to benzene. Our recent work on the naphthalene/Ni(111) system^21−23^ showed similar chemistry to benzene and naphthalene on other TM surfaces: upon heating, the initially planar naphthalene reacts by loss of two H atoms, and the C_10_H_6_ radical then tilts away from the surface. The change in the aromaticity of the rings, from sp^3^ back to sp^2^, as the π-system moves away from the surface can be seen in the frequency of the C–H stretches. Further heating leads to further dehydrogenation and the formation of graphitic and carbidic carbon. Above 700 K the carbon dissolves into the nickel surface. We have also investigated the effects of coadsorbed oxygen and sulfur^23,24^ on the adsorption and decomposition of naphthalene.

To our knowledge, there are no reports of studies investigating naphthalene on well-defined iron surfaces. The chemistry of iron surfaces involved in the Fischer–Tropsch synthesis (FTS) has been investigated in great detail, including recent ambient pressure XPS measurements. These studies have determined the chemical shifts of different hydrocarbon fragments and for carbon atoms in different binding sites on iron surfaces.^25−27^

In the present work, we examine the reaction of naphthalene on Fe(110) and the effect of surface oxygen. The goals of this detailed surface science study are to (i) compare the inherent reactivity of nickel and iron for tar reforming. (ii) Investigate how this is changed by adsorbed oxygen and carbon. (iii) Use this knowledge to try to explain the underlying factors contributing to the diverse results observed in the catalytic studies.^2−10^ We employed X-ray photoelectron spectroscopy (XPS) to study the surface adsorbates after annealing at different temperatures to follow the different steps of naphthalene dehydrogenation. Temperature-programmed desorption (TPD) was used to study the desorption products. Complementary experiments using sum frequency generation (SFG), and low-energy electron diffraction (LEED) were conducted to examine the initial adsorption and the structural changes induced by annealing and subsequent reactions. By examining the temperature-dependent desorption of hydrogen and CO, we can semiquantitatively evaluate the activity of the different surfaces. Additionally, a detailed comparison with the reaction on Ni(111) is presented.

## Experimental Methods

### X-ray Photoelectron Spectroscopy

The X-ray photoelectron spectroscopy (XPS) experiments were performed at the FinEstBeAMS materials and atmospheric science beamline at the MAX IV 1.5 GeV storage ring in Lund, Sweden. The solid-state end-station is optimized for ultraviolet and soft X-ray radiation with precisely controlled and widely variable parameters ideal for surface and interface science research, as described in detail in the original beamline publication.^28,29^ The end-station features in situ sample preparation and surface characterization. It is designed in a way that allows Ar-sputtering, a gas dosing system, a LEED setup, sample heating, and XPS experiments. The base pressure was 1.0 × 10^–10^ mbar in the preparation chamber and in the low 10^–10^ mbar range in the analysis chamber during experiments.

The sample was mounted on a sample holder made of molybdenum, secured with molybdenum screws and tantalum clamps. Heating was applied by resistive heating in the analysis chamber until 517 K and for higher temperatures in the preparation chamber to avoid outbound degassing material damaging the electron spectrometer. When the sample was not heated, the surface had a temperature of 298 K.

The Fe sample was cleaned through cycles of Ar^+^ sputtering at 840 K (1 kV, 10 mA at 1.5 × 10^–5^ mbar for 30 min). The quality of the surface was determined using LEED, showing a sharp hexagonal Fe(110) pattern and low background and proven by XPS scans on nitrogen, oxygen, sulfur, carbon, iron, potassium, and overview spectrum from 0 to 900 eV.

Pure naphthalene was purchased from Sigma-Aldrich and dosed onto the surface through a precision leak valve at 4.0 × 10^–8^ mbar. Doses are given in L (Langmuir) where 1 L equals 10^–6^ Torr × s. It takes ca. 10 L to form a saturated monolayer at room temperature.^21^ The time was started after the pressure in the preparation chamber exceeded 7.5 × 10^–9^ Torr (1 × 10^–8^ mbar) to a maximum pressure of around 3 × 10^8^ Torr (4 × 10^–8^ mbar) to get a dosing of around 10 L. The dosing was approximately calculated from the integral of the pressure-time curve. The naphthalene source was attached to the gas line before the experiments, and the line was baked out to achieve better background pressure. The purity of the naphthalene source was verified using a quadrupole mass spectrometer in the prep chamber after thoroughly degassing through the gas line.

**Figure 1:**
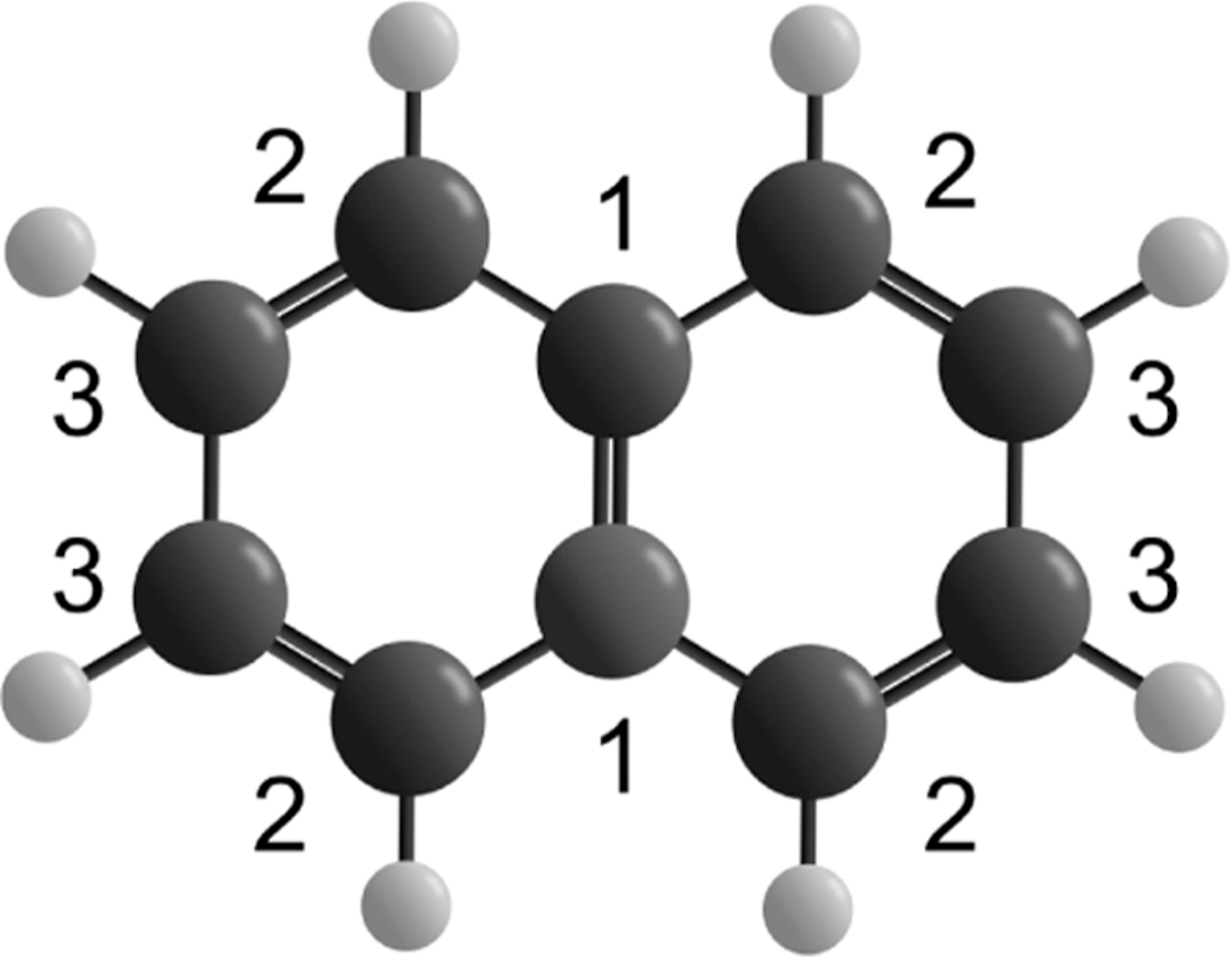
Naphthalene molecule with three chemically distinct carbon types.

For the C 1s spectra, a photon energy of 400 eV was used, and the settings in the beamline and analyzer were chosen to give a resolution of better than 150 meV. The binding energy scale was corrected to the Fermi level position measured after each scan. The spectra were fit using the LG4X python package^30^ and a skewed Voigt function for the peaks as provided by the python lmfit package. This peak shape gave better results in the peak tails than the skewed pseudo-Voigt function that was used on Ni(111).^23^ The asymmetry in the XPS peaks is due to vibronic structure. For gas-phase benzene^31^ and naphthalene^32^ the line-shapes have been analyzed in detail, finding less broadening of the C1 compared to the C2/C3 XPS peaks in naphthalene (see Figure 1), where excitation of C–H modes is more likely. On a surface, the vibronic structure will be different and will also change during the reactions. The peak parameters were numerically extracted due to the shift introduced by the skew.

### Temperature-programmed Desorption and Sum Frequency Generation Spectroscopy

The temperature-programmed desorption (TPD) and sum frequency generation (SFG) experiments were performed in a UHV chamber equipped with a QMS (quadrupole mass spectrometer), a LEED setup, and an ion gun for sputtering. The base pressure of the chamber is in the low 10^–10^ mbar range. Temperature control of the sample was achieved using liquid nitrogen cooling, resistive heating and e-beam heating.

Resistive heating at a fixed ramping rate of 100 K/min was used for the TPD experiments, and masses/charge (*m*/*z*) of 2, 18, 28, 44, and 128 were monitored using the QMS.

SFG provides a means to obtain surface-sensitive vibrational spectra by measuring the mixing of two laser fields at the surface.^33−35^ The probability of SFG is changed by the presence of oscillators at the surface that are resonant with one of the laser fields. Here, a broad-band IR pulse overlaps in frequency with vibrational modes of adsorbed species and the resonances can be seen as changes in the SFG intensity. From symmetry considerations, vibrational modes must be both IR- and Raman-active to be observed in the SFG spectrum and on a metal surface the vibration must also change the dipole moment perpendicular to the surface as the parallel components are effectively screened by the polarizable conduction band electrons. On metal surfaces, the free electrons can also contribute to SFG, leading to a broad, nonresonant, background (NRB).^35^

To determine the positions and intensities of the resonances, the spectra were fitted using the following equation for the second order susceptibility χ^(2)^, on which SFG intensity is quadratically dependent^35^
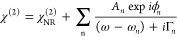
1where χ_NR_^(2)^ is the contribution of the nonresonant background, and *A*_*n*_, ϕ_*n*_, ω_*n*_ and Γ_*n*_ are the amplitude, phase, frequency and width of the *n*th resonance. The nonresonant background (NRB) is measured on the clean surface and changes only in amplitude upon absorption. A change in the NRB induced by sample movement during the heating series was accounted for by interpolation between the low and high temperature NRB. All fitted amplitudes used in this paper were robust throughout different approaches in treating the NRB temperature changes.

The setup is equipped with a mode-locked Ti/sapphire laser system producing pulses of ≤50 fs width at a wavelength of 800 nm and a repetition rate of 1 kHz. Pulses are split into a narrow-bandwidth 800 nm pulse and a broadband, tunable wavelength mid-IR pulse generated using a tunable optical parametric amplifier and a noncollinear difference frequency generator, which was centered around 3200 nm (C–H stretching region). The pulses are temporally and spatially overlapped on the sample surface for sum frequency generation and the resulting light is analyzed in a spectrometer using an iCCD camera with a resolution of ∼14 cm^–1^. For temperature-dependent SFG, resistive heating with a ramping rate of 6 K/min was used and spectra were recorded continuously while heating, with a temperature resolution of 3 K.

## Results and Discussion

### Adsorption of Naphthalene on Fe(110) and Dehydrogenation

The naphthalene molecule has three distinct carbon atoms, which will be referred to as C1, C2 and C3 as depicted in Figure 1. To study the initial adsorption of naphthalene and the dehydrogenation upon heating, SFG and TPD were used.

**Figure 2:**
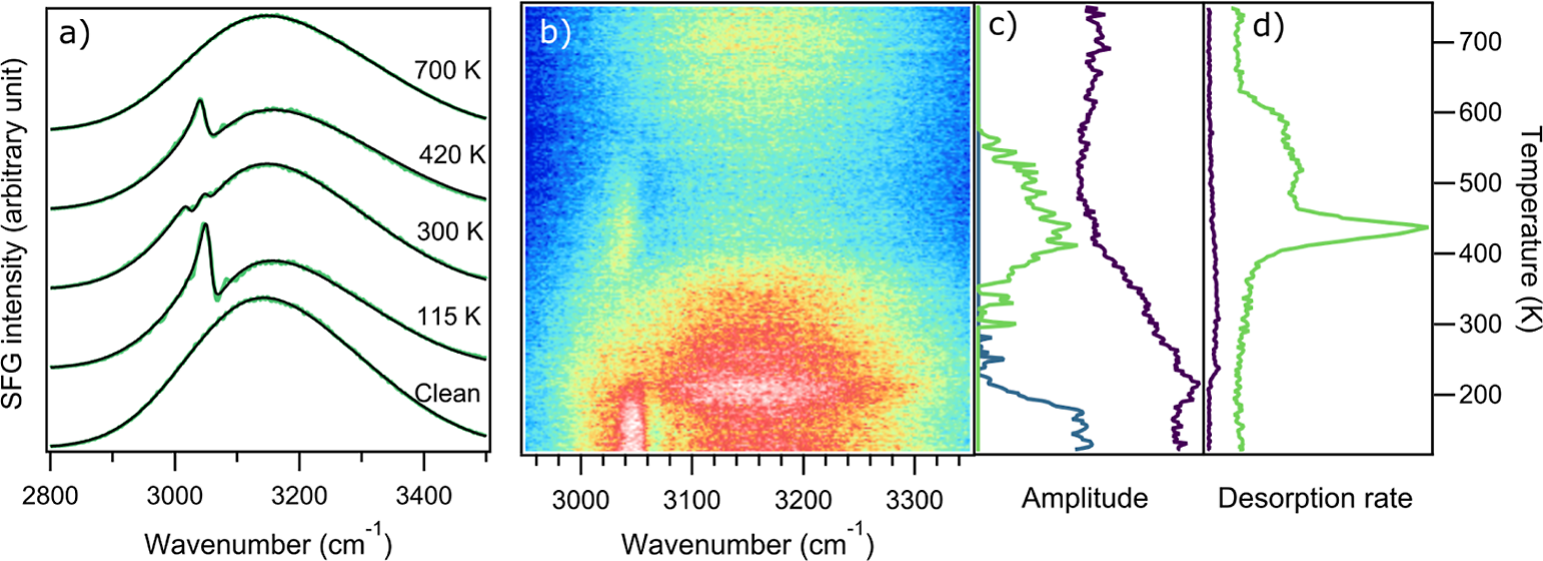
Heating of 10 L naphthalene dosed on clean Fe(110) studied with SFG and TPD: (a) SFG spectra of a clean surface and 10 L naphthalene dosed at 115 K and flashed to different temperatures (green) and corresponding fits (black), (b) false color plot of temperature dependent SFG series with a heating rate of 6 K/min, where blue regions indicate low and white regions high intensities, (c) total SFG intensity (purple) and fitted amplitudes of multilayer (blue) and partially dehydrogenated (green) resonances, (d) temperature dependent desorption rates of *m*/*z* 2 (green) and 128 (purple).

Figure 2 shows temperature-dependent SFG and TPD measurements for 10 L naphthalene dosed on Fe(110). SFG spectra accumulated at 115 K after flashing the sample to different temperatures are shown in Figure 2a to illustrate the specific resonances that are observed. The nonresonant background (NRB) of the clean Fe(110) sample at 115 K is shown at the bottom of Figure 2a. Figure 2b shows a false color plot of a temperature-dependent SFG series in the C–H vibrational frequency region taken during sample heating, where blue indicates low and white high intensity. Each horizontal line represents one recorded spectrum. Following dosing and up to 200 K there is a single strong resonance around 3055 cm^–1^. Between 250 and 350 K two weak resonances at 3023 and 3051 cm^–1^ are visible. At 350 K a resonance appears at 3045 cm^–1^. The intensity of this band increases up to a maximum between 400 and 550 K and then disappears above 580 K. Above 600 K all resonances disappear and only the nonresonant background is observed at 700 K (see Figure 2a). The decrease of the NRB can also be observed in the purple line in Figure 2c, which represents the total SFG intensity, mostly governed by the NRB.

Figure 2d shows TPD spectra for a 10 L dose of naphthalene with a ramping rate of 100 K/min for 2 (green) and 128 *m*/*z* (purple). Naphthalene desorption starts at 200 K and continues in a broad, weak feature up to ca. 500 K. There are two main features in the H_2_ TPD, with a strong, narrow peak centered at 450 K and a broad higher temperature peak centered around 550 K. Between 300 and 400 K there is a weak shoulder, showing some dehydrogenation starts at lower temperatures.

Using the combination of the SFG and TPD results we interpret the surface reactions and species in the following way: Initially, after naphthalene adsorption, a strong resonance of the naphthalene multilayer is observed at 3055 cm^–1^ in the 115 K spectrum. We attribute this strong SFG signal to the C–H vibrations of naphthalene molecules in the multilayer. This is supported by comparison with NIST IR reference spectra,^36^ matrix isolation IR spectroscopy of naphthalene, where intense bands due to C–H vibrations were observed at 3065 and 3078.2 cm^–1^,^37^ and the C–H stretches of multilayer naphthalene on Ni(111).^22^ We note that these different methods and environments lead to changes in the frequencies and relative intensities of the active modes. This resonance starts to decrease rapidly at 200 K and has disappeared by 220 K, coinciding with naphthalene desorption in the TPD spectrum. Above 220 K, chemisorbed naphthalene remains on the surface. The weak SFG response of this layer is presumably due to the molecules lying with the rings parallel to the surface, similar to the case for benzene/Fe(110)^14^ with the C–H bonds only slightly out of the molecular plane. The presence of two resonances suggests a disordered structure with more than one type of binding site or species for naphthalene on Fe(110). At 380 K a new resonance at 3045 cm^–1^ appears in the SFG spectrum, which grows in intensity until 400 K. The first H_2_ desorption peak in the TPD occurs at the same temperature, consistent with the new resonance being due to partially dehydrogenated naphthalene (C_10_H_6_) which is tilted away from the surface, similar to the case on Ni(111)^22^ or for benzene.^13,17,18^ The increased intensity may be due both to the change in the orientation of the C–H bonds in the molecules and an increased degree of order on the surface.

At higher temperature, dehydrogenation continues, seen in the continued desorption of H_2_ in TPD and the disappearance of the C–H resonance in the SFG spectrum. Above 600 K, no resonances are visible and the NRB is slightly decreased compared to the clean surface due to surface carbon from the naphthalene decomposition changing the polarizability of the surface.

The room temperature reactivity observed in the H_2_ TPD can be attributed to reactions on step and defect sites. This argument is supported by the fact that by repeating the TPD with a rough (sputtered but not annealed) surface, the room temperature desorption peak is drastically increased.

**Figure 3:**
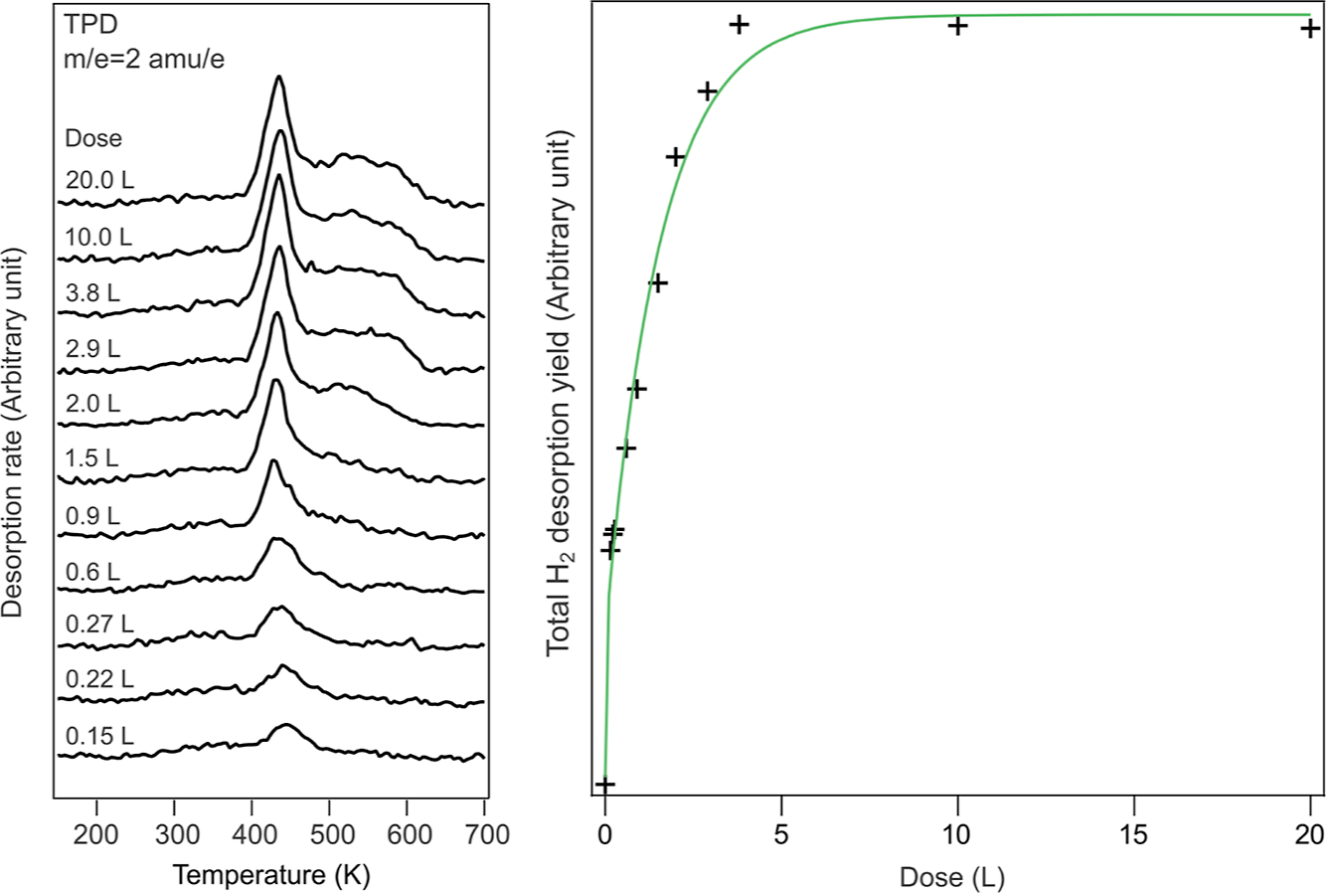
Temperature dependent desorption of H_2_ after dosing naphthalene onto Fe(110) at different naphthalene doses (left) and total H_2_ yield (integrated area) as a function of naphthalene dose (right), with a heating rate of 100 K/min. The green fit was included to serve as a visual reference.

We have investigated the effects of surface coverage on the reactivity of naphthalene using TPD. Figure 3 shows the temperature dependent desorption of H_2_ following different doses of naphthalene on Fe(110). At first, the 450 K-peak grows in with increasing dose, followed by the development of the broad high-temperature peak. This suggests that at low coverage the activation barriers are similar for all of the dehydrogenation steps and it is only with higher coverage, above around 1.5 L exposure, that the later reaction steps become slower. The reason(s) for this change in activity with increasing naphthalene coverage are currently not clear. At naphthalene doses above 3.8 L, the TPD spectra remain largely unchanged. This is also true for the total hydrogen signal, which is plotted as a function of naphthalene dose on the right side of Figure 3. The hydrogen production levels off around 3–4 L. This can be explained by the saturation of the naphthalene monolayer, since any multilayer naphthalene desorbs below room temperature where no dehydrogenation occurs.

**Figure 4:**
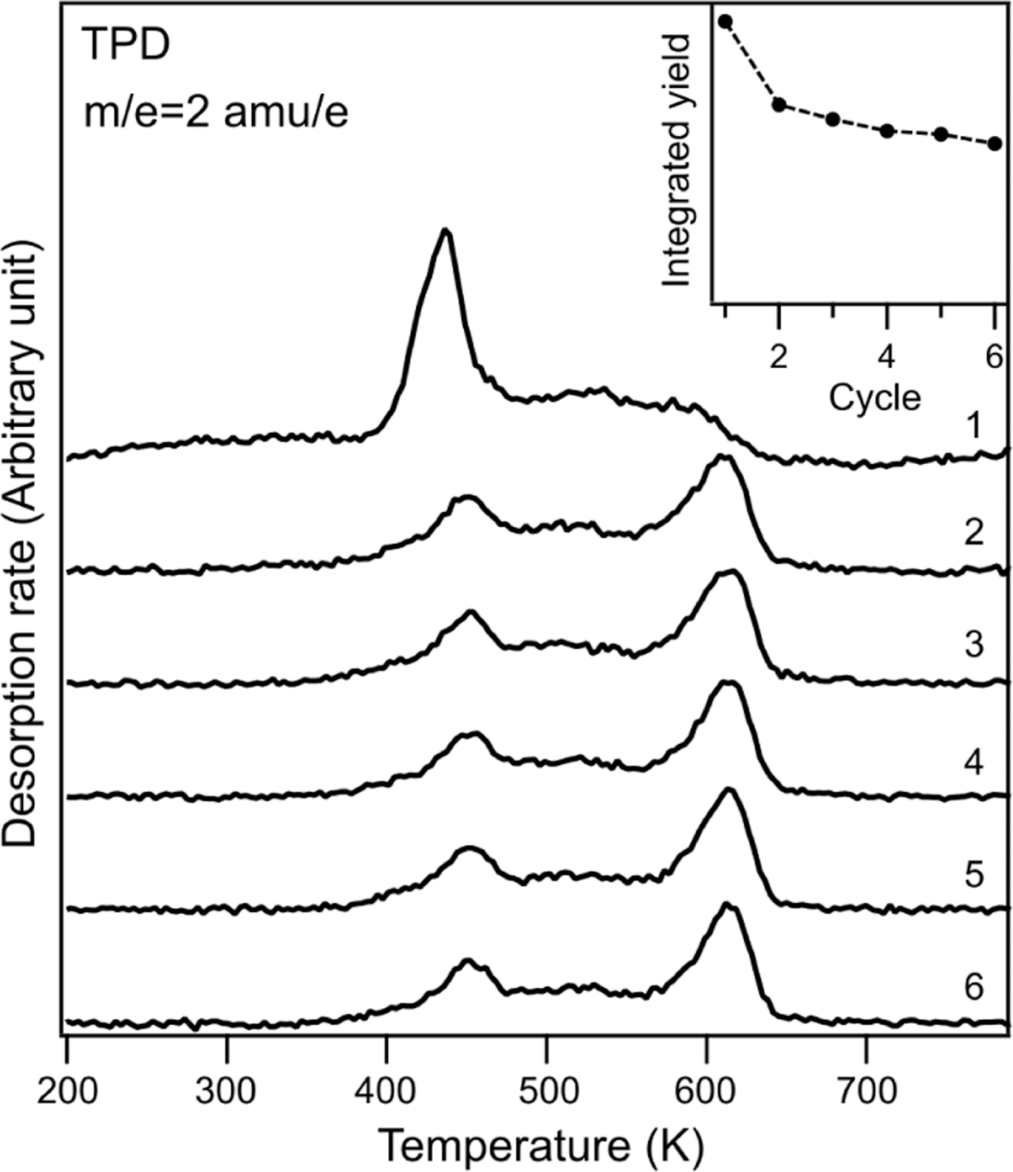
Hydrogen desorption from naphthalene on Fe(110), multiple dosing cycles without intermittent surface cleaning. The inset shows the integrated yield vs dosing cycles.

Passivation caused by surface carbon can be observed in the temperature dependent hydrogen desorption of consecutive naphthalene dosing and annealing cycles, as depicted in Figure 4. A significant drop (to about 60% of the hydrogen yield) is observed after the first cycle. The high temperature desorption peak becomes narrower and shifts to higher temperatures as the catalytic activity declines. This is followed by a region of markedly slow decline, where the desorption temperatures do not change at all and total H_2_ production decreases only very little each cycle. Thus, the activity seems to be relatively stable over a range of carbon coverage with slow decline due to suppression of naphthalene adsorption.

**Figure 5:**
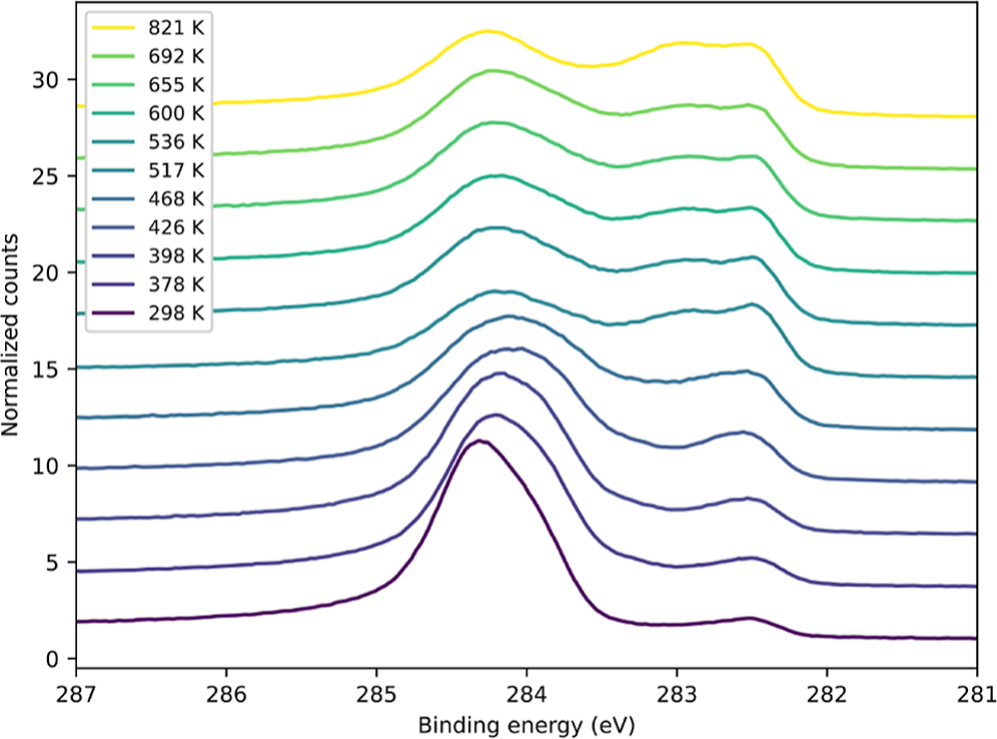
Series of C 1s XPS spectra of 10 L naphthalene dosed on Fe(110) after annealing to different temperatures. The photon energy was 400 eV.

To gain more information about the carbon species on the surface during the naphthalene dehydrogenation, the reaction was studied with XPS. Figure 5 shows a series of C 1s spectra after dosing 10 L naphthalene onto the Fe(110) surface at 298 K and annealing to different temperatures. At room temperature, the naphthalene features can be observed as a broad peak centered around 284.3 eV. This peak is not well resolved, and the typical “naphthalene peak shape”^24,32^ with one intense peak and a higher BE shoulder representing C2/C3 and C1, respectively (see Figure 1), is not present. The additional small peak at 282.5 eV observed at room temperature is also detected on the clean surface and represents segregated or chemisorbed carbon.^38^ As mentioned earlier, hydrogen desorption begins around 300 K and the production of surface carbon can be expected as a result of the surface reaction. With increasing temperature, the peaks in the “chemisorbed carbon” region become more dominant, and additional intensity emerges between 283.0 and 283.6 eV, which can be attributed to carbidic carbon.^27^ The naphthalene peak diminishes during heating, leaving some C=C carbon on the surface even after full dehydrogenation. The amount of surface carbon does not decrease further at very high temperatures, but starts to increase due to segregation from the bulk. It is not possible to dissolve all of the carbon into the crystal bulk upon heating.

**Figure 6:**
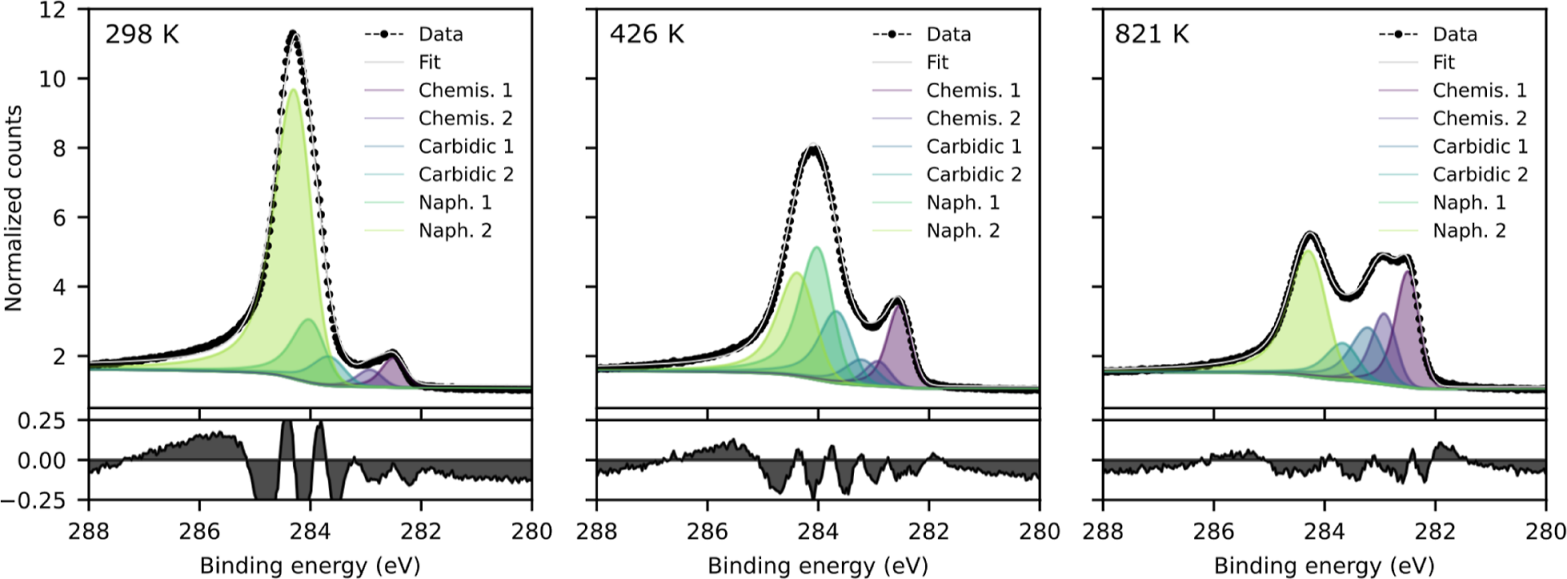
Example fits of the C 1s XPS spectra of 10 L naphthalene dosed on Fe(110) at 298, 426, and 821 K.

To capture the temperature trends of the different carbon species, the spectra were fit using asymmetric Voigt functions. Figure 6 shows three example fits at 298, 517, and 821 K. It is not possible to distinguish the contributions of the carbon atoms C2/C3 with hydrogen and the C1 atoms without hydrogen (see Figure 1). Two peaks were used to fit the naphthalene contribution as shown in Figure 6. However, due to limited resolution these peaks are not based on specific carbon species, and do not allow for their differentiation. As mentioned previously, TPD and SFG results show that dehydrogenation already begins at room temperature, and that the naphthalene monolayer appears to be more disordered on Fe(110) than what was observed on Ni(111). The peaks representing the naphthalene, partially dehydrogenated naphthalene, C_*x*_ H_*y*_ and C=C fragments were therefore chosen by trial and error for consistency throughout the temperature series, and are referred to as “naphthalene” peaks in the fits. Additionally, two different peaks for carbidic carbon were employed, which have been previously described by Shipilin et al. as carbides with two different coordination sites for the carbon atoms.^27^ In our spectra, these peaks are found at 283.7 and 283.3 eV, respectively. Two peaks were also used for the chemisorbed carbon, at 282.5 and 282.9 eV. These were observed as a peak and shoulder in a C 1s spectrum taken from the surface without naphthalene but with residual carbon present. Two different binding energies concurrent with these peaks were found for segregated/chemisorbed carbon in the literature.^25,38,39^ The fits are notably less accurate at low temperatures (room temperature up to around 400 K), likely due to the high binding energy tail. It is possible that there is some CO on the surface due to the dosing procedure, which would cause an increased intensity in the tail above 285 eV. This then disappears when the CO desorbs at around 400 K.

**Figure 7:**
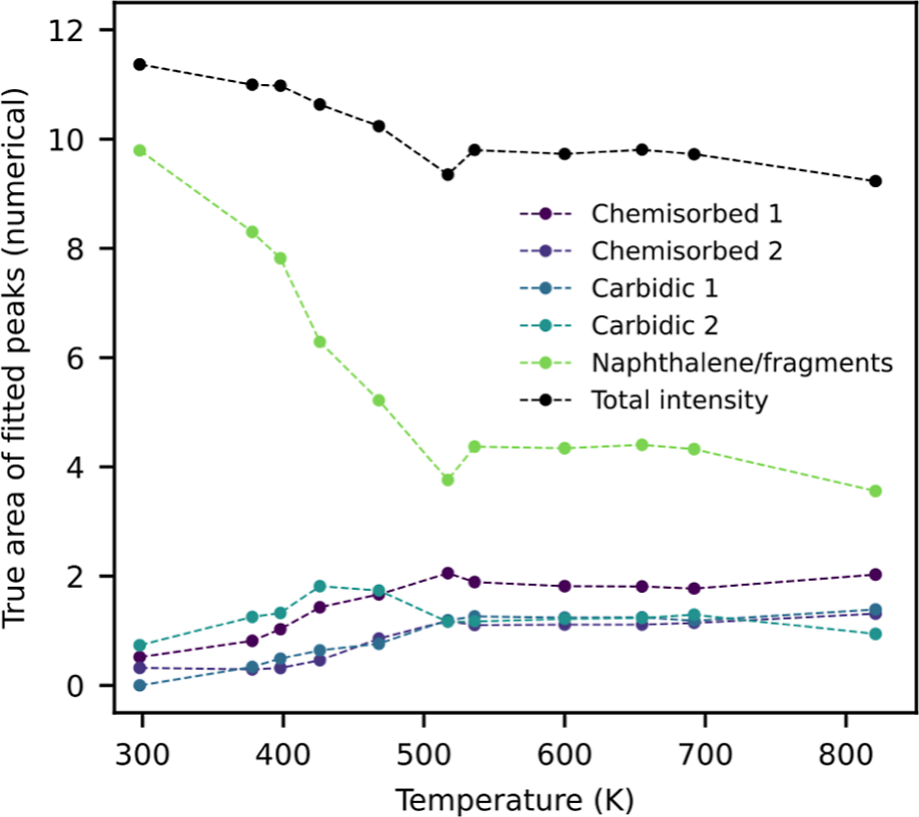
Temperature trends of the fitted XPS peak areas for naphthalene on Fe(110). The two naphthalene peaks were summed together since they overlap too strongly to distinguish individual carbon species. Black line: total C 1s intensity.

The temperature trends of the fitted peak areas are depicted in Figure 7. The two peaks representing naphthalene and the fragments resulting from naphthalene decomposition are summarized into a single intensity, since they contain multiple carbon species and lack sufficient resolution to be meaningfully separated. There is an uncertainty in the quantitative peak areas due to the large overlap of the peaks, mainly in distinguishing high binding energy carbide from naphthalene fragments and low binding energy carbide from the chemisorbed peak. However, the temperature trends are consistent even with different starting parameters for the fitting procedure. The initial decay of the naphthalene peak, together with the rise of the carbidic and chemisorbed carbon peaks, can be clearly observed. After around 500 K, the naphthalene intensity stabilizes. It is expected that the progressing dehydrogenation results in either graphitic carbon or C=C fragments. Some publications observe graphitic carbon on Fe(110) at a binding energy of around 284.5 eV or higher.^25,27^ However, ordered graphene layers, such as in HOPG, are usually observed around 284.1–284.2 eV, with some shift expected on the metal surface.^40^ Wiltner and Linsmeier distinguished two types of graphitic carbon on Fe(110):^26^ “ordered” graphite at 284.2 eV and “disordered” graphite at 285.1 eV. Thus, the peak remaining around 284.2 eV after full dehydrogenation is likely caused by ordered graphene-like residues of the aromatic ring. In the high temperature spectra (see the 821 K spectrum of Figure 6), the remaining naphthalene fragment peak has a binding energy of around 284.3 eV. It is not possible to unambiguously determine the chemical nature of the carbon contributing to this peak, but it likely involves C=C fragments remaining after dehydrogenation and ring cleavage, as well as some “disordered” graphitic carbon or sp^3^ carbon contributing to the increase in binding energy at high temperature.

The black line in Figure 7 shows the total intensity of the C 1s peaks as a function of annealing temperature. An initial decrease of about 18% is observed up to a temperature of 500 K, followed by a slight increase around 550 K. Segregation from the bulk has been observed on a Fe(100) surface at this temperature in previous experiments in our group. No carbon containing gas phase products were detected during TPD measurements (monitored carbon species: *m*/*z* 14 (CH_2_), 16 (CH_4_), 27 (C_2_ H_3_), 28 (CO), 30 (CH_2_O, CHOH), 31 (CH_3_O, CH_2_OH), 32 (CH_3_OH)). Thus, it is likely that some dissolution into the bulk occurs initially up to a surface temperature of around 550 K, after which the equilibrium shifts toward surface segregation.

### Influence of Oxygen

**Figure 8:**
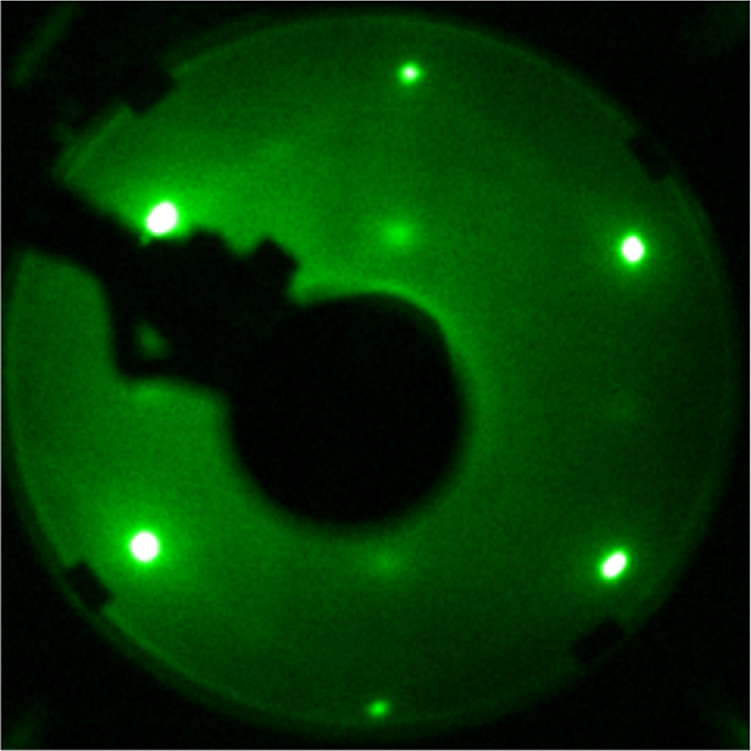
LEED pattern of the c(2 × 2) oxygen overlayer on Fe(110), recorded with a beam energy of 122 eV, taken after dosing 1.5 L of O_2_.

In catalytic studies, the reactivity of iron catalysts toward oxygen and the lowered reactivity of oxidized/oxygen passivated iron surfaces are often cited as a reason for the lower performance of iron compared to nickel catalysts.^4,6^ We studied the effect of a low oxygen dose, 0.5 L for XPS and 1.5 L at the TPD/SFG setup. In both cases LEED imaging was used to confirm the formation of a c(2 × 2) overlayer previously described in the literature,^41^ which corresponds to a coverage of 0.25 ML. The LEED pattern from the surface after dosing 1.5 L oxygen, recorded with a beam energy of 122 eV, is depicted in Figure 8.

**Figure 9:**
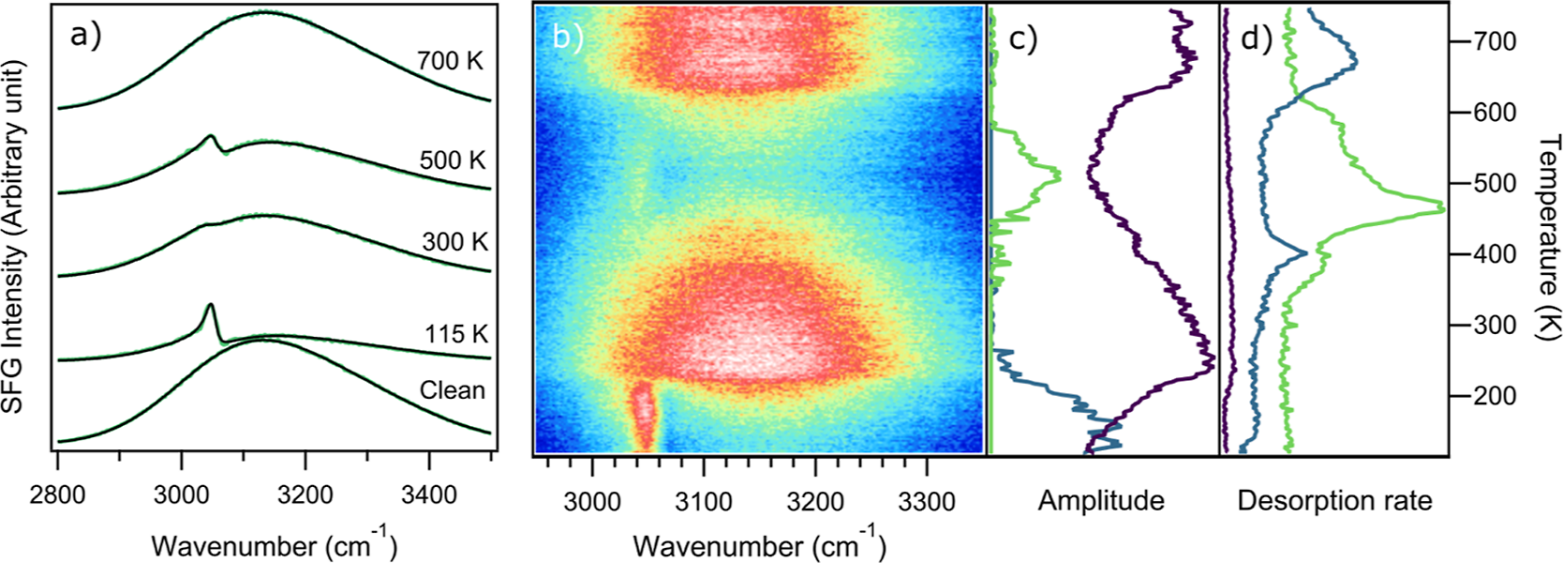
Heating of 10 L naphthalene dosed on c(2 × 2)O/Fe(110) formed by exposure of Fe(110) to 1.5 L O_2_ studied with SFG and TPD: (a) SFG spectra of a clean surface and 10 L naphthalene on 1.5 L O_2_ dosed at 300 K and flashed to different temperatures (green) and corresponding fits (black), (b) false color plot of temperature dependent SFG series with a heating rate of 6 K/min, where blue regions indicate low and white regions high intensities, (c) total SFG amplitude (purple) and fitted amplitudes of multilayer (blue) and partially dehydrogenated (green) resonances, (d) temperature dependent desorption rates of *m*/*z* 128 (purple), 28 (blue) and 2 (green).

Figure 9 shows the results of SFG and TPD measurements of the heating of 10 L of naphthalene on c(2 × 2)O/Fe(110). In Figure 9a, individual SFG spectra of 10 L naphthalene dosed after 1.5 L oxygen at different temperatures (naphthalene dosed at 115 K after dosing O_2_ at 300 K) are depicted alongside the NRB on the clean Fe(110) surface. Figure 9b shows the false color plot of the SFG series measured during annealing. In the presence of oxygen, the NRB is suppressed compared to the naphthalene multilayer on clean Fe(110) (115 K spectrum in Figure 9a), which is as expected for the additional oxygen layer. The changes in the SFG spectra upon annealing are similar to those seen of the clean Fe(110) surface, with a few notable differences: In the 300 K spectrum, we only observe one weak resonance around 3040 cm^–1^ which is weaker than those observed without oxygen. The appearance of the partially dehydrogenated resonance occurs at slightly higher temperatures, the green line in Figure 9c shows the temperature dependence of the intensity of the resonance obtained from fits to the spectra. The observed resonance frequencies of the three surface species we assume are present are more similar on the c(2 × 2)O/Fe(110) surface than on the clean surface or Ni(111).^22^ The NRB intensity is greater after full dehydrogenation, closer to that of the clean Fe(110) surface than that of the c(2 × 2) oxide or the carbon covered surface obtained without oxygen. This is also seen in the increase of total intensity (see purple line in Figure 9c).

In the TPD spectra, the desorption of hydrogen (green line in Figure 9d) starts around 470 K, somewhat higher than without oxygen (around 440 K). Additionally, the desorption of CO is observed, with peaks at 400 and 600 K. The CO peak at 400 K is due to background gas in the vacuum chamber sticking to the surface during oxygen dosing.

From these results, we conclude that the reaction mechanism for dehydrogenation of naphthalene on c(2 × 2)O/Fe(110) is similar to that on the clean surface, i.e. the multilayer desorbs without reacting, the monolayer molecules lie flat on the surface and then tilt up following the loss of two H atoms. However, the chemisorbed/monolayer naphthalene appears to be less activated on the oxide than on the clean surface. This can be seen in the both the resonance frequency and intensity of the SFG spectrum of the monolayer naphthalene, where the C–H stretch frequency is unchanged compared to the multilayer molecules. The low intensity of the resonance implies the C–H bonds are lying almost parallel to the metal. The lower degree of activation is also supported by the higher temperature needed before the SFG resonance starts to increase in intensity and to induce H_2_ desorption in the TPD measurement. If we assume that the Arrhenius prefactors for the rate-determining step are the same on both surfaces, and that the changes we see in the experiments occur for the same surface reaction rates, we can estimate the barrier on the oxide to be about 7% higher
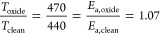
2

The higher intensity of the NRB at high temperature, due to the higher free electron density of the clean surface compared to the oxide surface, and the observation of CO production in TPD shows that surface carbon produced by naphthalene decomposition can react with the surface oxygen to complete a catalytic cycle. The CO formation starts at around 600 K (see blue line in Figure 9d), and as a consequence leaves the surface cleaner than after naphthalene decomposition without oxygen. This demonstrates that iron can be used in a catalytic reforming cycle in a suitable oxidizing atmosphere, with partial oxidation of carbon to valuable CO.

**Figure 10:**
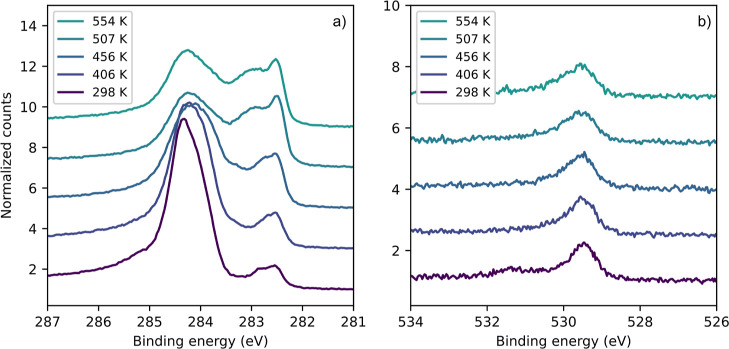
XPS series of (a) C 1s and (b) O 1s spectra of 10 L naphthalene dosed on Fe(110) + 0.5 L oxygen (c(2 × 2) oxygen overlayer) after annealing to different temperatures. The photon energy was 400 and 650 eV for the C 1s and O 1s, respectively.

Figure 10 shows a XPS annealing series of C 1s and O 1s spectra of 10 L naphthalene on Fe(110) with 0.5 L oxygen dose up to 554 K. The C 1s spectra (see Figure 10a) are similar to those observed for naphthalene on clean Fe(110). With oxygen, a small shoulder appears on the high binding energy side at room temperature due to CO species. In the O 1s spectra (see Figure 10b), the main peak around 529.5 eV is due to oxygen bonded to Fe(110).^42,43^ A small additional peak at around 531.4 eV is seen at room temperature, which is due to adsorbed CO species at temperatures below 406 K. The intensity of the O 1s peaks is slightly reduced at 554 K likely due to the start of reactions with the surface carbon.

**Figure 11:**
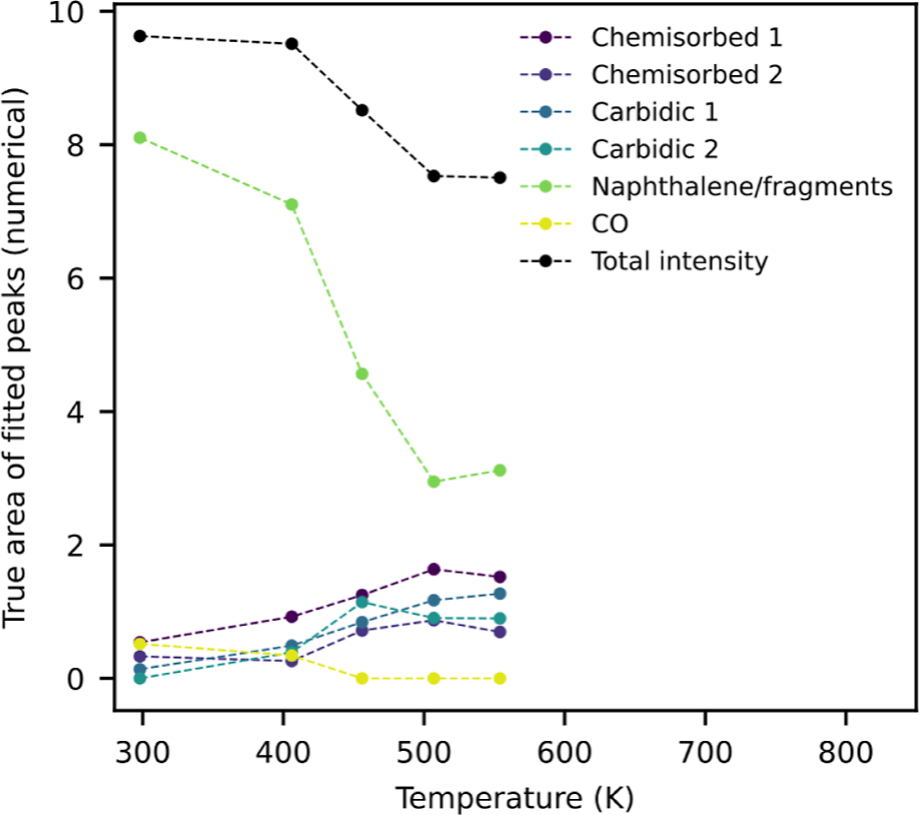
Temperature trends of the fitted XPS peak areas for naphthalene on Fe(110) + 0.5 L oxygen dosed. The two naphthalene peaks were summed up since they overlap too strongly to distinguish individual carbon species. Black line: total C 1s intensity.

A simpler comparison between the oxygen covered and clean Fe(110) is possible from the fitted peak areas. Figure 11 shows the temperature trends of the fitted peaks, with the naphthalene peaks summed due to the difficulty in distinguishing individual species. For the data set with oxygen, a CO peak was added, which is visible only at RT and 406 K and was set to zero for the higher temperature fits, as it is not present any longer. The overall trend of the peak areas is largely similar to that observed on the clean surface. The highest temperature measured in the presence of oxygen due to experimental challenges was 554 K, too low to observe the reaction of the surface carbon with the oxygen layer to gaseous CO.

To investigate the evolution of the surface after the reaction of the c(2 × 2) oxygen overlayer with the saturated naphthalene monolayer, a series of TPD measurements was conducted with redosing both oxygen and naphthalene in between the cycles.

**Figure 12:**
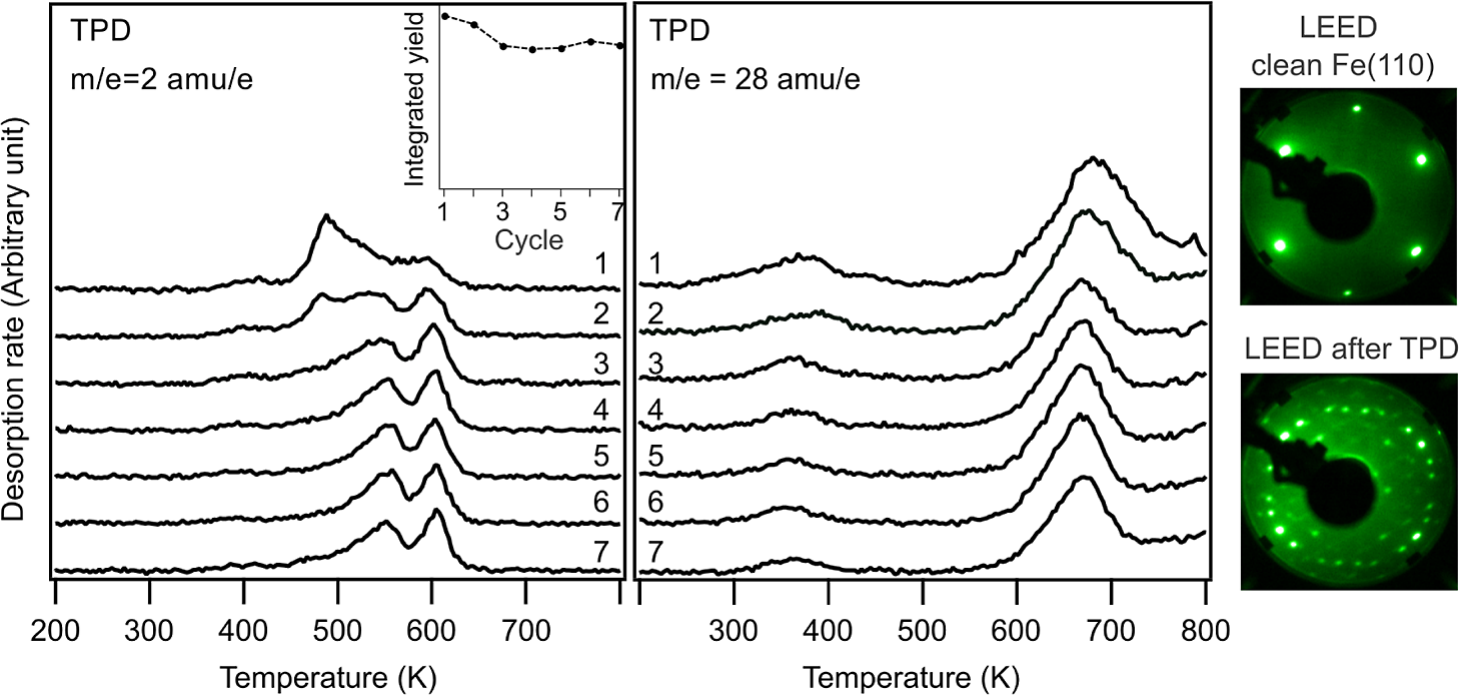
Series of hydrogen (left) and CO (right) desorption traces after redosing 1.5 L O_2_ and 10 L naphthalene for multiple cycles. The inset shows the integrated yield vs dosing cycles. Far right: LEED images, recorded with a beam energy of 122 eV, of the clean Fe(110) and after dehydrogenation when steady state (supported by TPD) is reached. The TPD spectra were recorded by using a heating rate of 100 K/min.

Figure 12 shows the series of hydrogen (left) and CO (right) desorption spectra. After the first cycle, hydrogen desorption shifts to higher temperature, with two clear peaks emerging at around 540 and 600 K. After three cycles, no more changes are observed in the hydrogen desorption, indicating that a steady state is reached. Hydrogen production only decreases by about a fifth compared to the clean surface (see inset). No significant changes were observed in CO desorption, which only occurs at elevated temperatures. The surface in this “steady state” regime has a consistent amount of surface carbon, which can be seen in the LEED images to the right, recorded with a beam energy of 122 eV. The LEED after the TPD cycles shows the carbon ring pattern in addition to the (1 × 1) spots of the Fe(110) surface.

**Figure 13:**
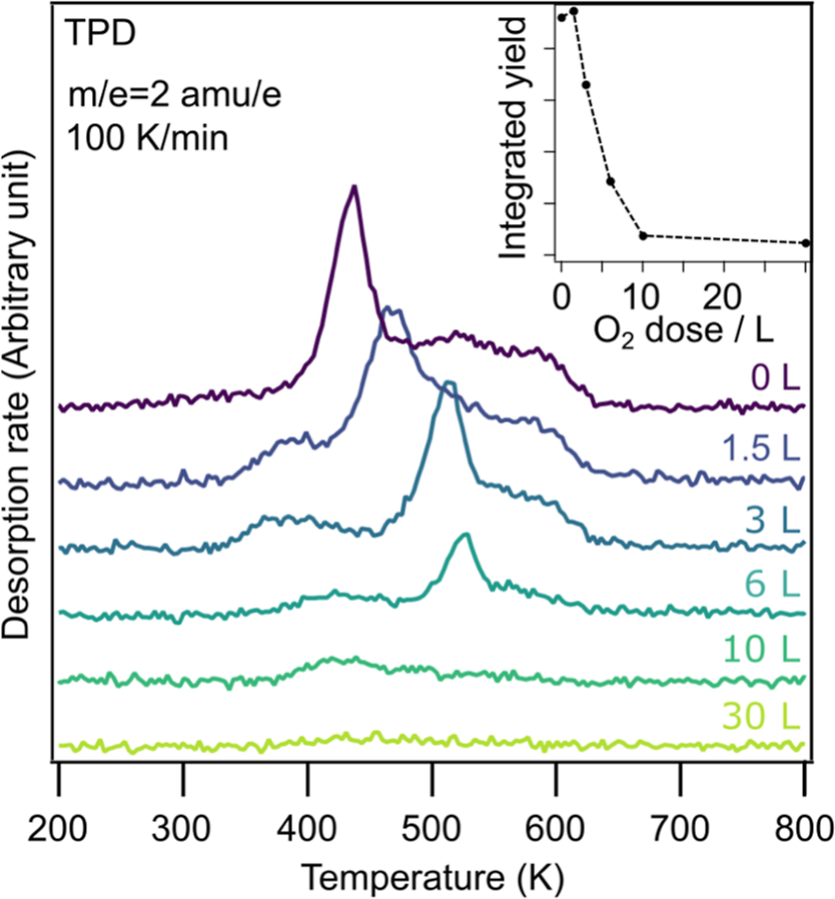
Thermal desorption of hydrogen from Fe(110) with different doses of oxygen. The inset shows the total H_2_ yield.

Figure 13 shows the change in the hydrogen desorption spectrum with increasing oxygen dose, as well as the relationship between total H_2_ production and oxygen dose (see inset). Up to a dose of around 3 L, the main hydrogen desorption peak shifts to higher temperature, and the low temperature shoulder grows into a more pronounced peak at around 400 K. In this dose region, the c(2 × 2) overlayer corresponding to 0.25 ML oxygen coverage should be observed.^41^ With increasing dose, which would correspond to what is usually referred to as the “complex” overlayer structure,^41^ a strong decrease of the peaks occurs. At 10 L dosed, only a small, broad peak at around 420 K is left, which further declines for 30 L, where no H_2_ formation is seen. The onset of FeO formation is expected to be between 10 and 30 L.^41^ This trend can be seen more clearly in the total hydrogen yield, which shows a sharp decline up to 10 L. Notably, the data point at 1.5 L is slightly higher than the 0 L value. While the increase is within the error and therefore may not be a real effect, it clearly shows that the drastic decline begins after 1 L.

### Comparison with Ni(111)

The trends observed in the SFG spectra for naphthalene adsorption on Fe(110) closely resemble those seen on Ni(111):^22^ there is one distinct resonance of the multilayer naphthalene around 115 K at 3055 and 3057 cm^–1^, for Fe(110) and Ni(111) respectively. At around 300 K the spectra for the two metals differ, indicating differences in monolayer adsorption. There are two weak resonances for Fe(110), at 3023 and 3051 cm^–1^, but only one for Ni(111) at 3003 cm^–1^, caused by a flat adsorption geometry with angled hydrogen atoms leading to a loss of the sp^2^ hybridization and an increase of the sp^3^ characteristics.^22^ The higher vibrational frequency and very low intensity of the monolayer naphthalene resonances on iron suggest that the carbon atoms have more sp^2^ character than on Ni(111). The weak, broad peak with multiple resonances points to more disordered naphthalene adsorption at room temperature. A plausible explanation is the difference in room temperature reactivity caused by defect sites and steps. The room temperature reactivity increases strongly on a rough surface, while on Ni(111), surface roughness was shown to have only a minor effect on dehydrogenation.^21^ However, to elucidate the exact adsorption geometry, microscopy or angle resolved X-ray absorption measurements would be necessary. Around 400 K, strong resonances appear for the partially dehydrogenated molecule, at 3045 (iron) and 3057 cm^–1^ (nickel), respectively. This resonance appears over a slightly larger temperature range for Fe(110), but otherwise the trend is very similar.

The temperature-dependent hydrogen desorption behavior on Fe(110) is similar to the one previously observed on Ni(111).^21^ In both cases, a prominent low temperature peak emerges around 450 K, followed by a broad, higher-temperature peak between approximately 500 and 600 K. On Fe(110), the low temperature peak is slightly narrower than that on Ni(111), while the broader high temperature peak on both surfaces appears to comprise multiple unresolved features.

The low temperature peak accounts for roughly 30% of the total hydrogen production and, as with Ni(111), likely corresponds to the cleavage of the first two hydrogen atoms from the naphthalene molecule, along with potential reactions at steps and defects. On Ni(111), it was found that the most favorable reaction pathway involves the abstraction of hydrogen atoms from neighboring C2 sites.^22^ The onset of the first hydrogen peak coincides with the formation of the partially dehydrogenated resonance observed in the SFG measurement (as shown in the 420 K plot in Figure 2a), which is the main SFG feature during dehydrogenation. The high temperature H_2_ desorption peak corresponds to the cleavage of the remaining hydrogen atoms. However, similar to Ni(111), the SFG resonance of the partially dehydrogenated molecule (see green line in Figure 2c) disappears prior to complete dehydrogenation. This loss is attributed to progressive C–C bond cleavage, leading to hydrocarbon fragments on the surface that are not detectable by SFG, likely due to the orientation of the C–H bonds. Overall, the Fe(110) surface exhibits comparable activity toward aromatic dehydrogenation as observed for the Ni(111) surface.

**Figure 14:**
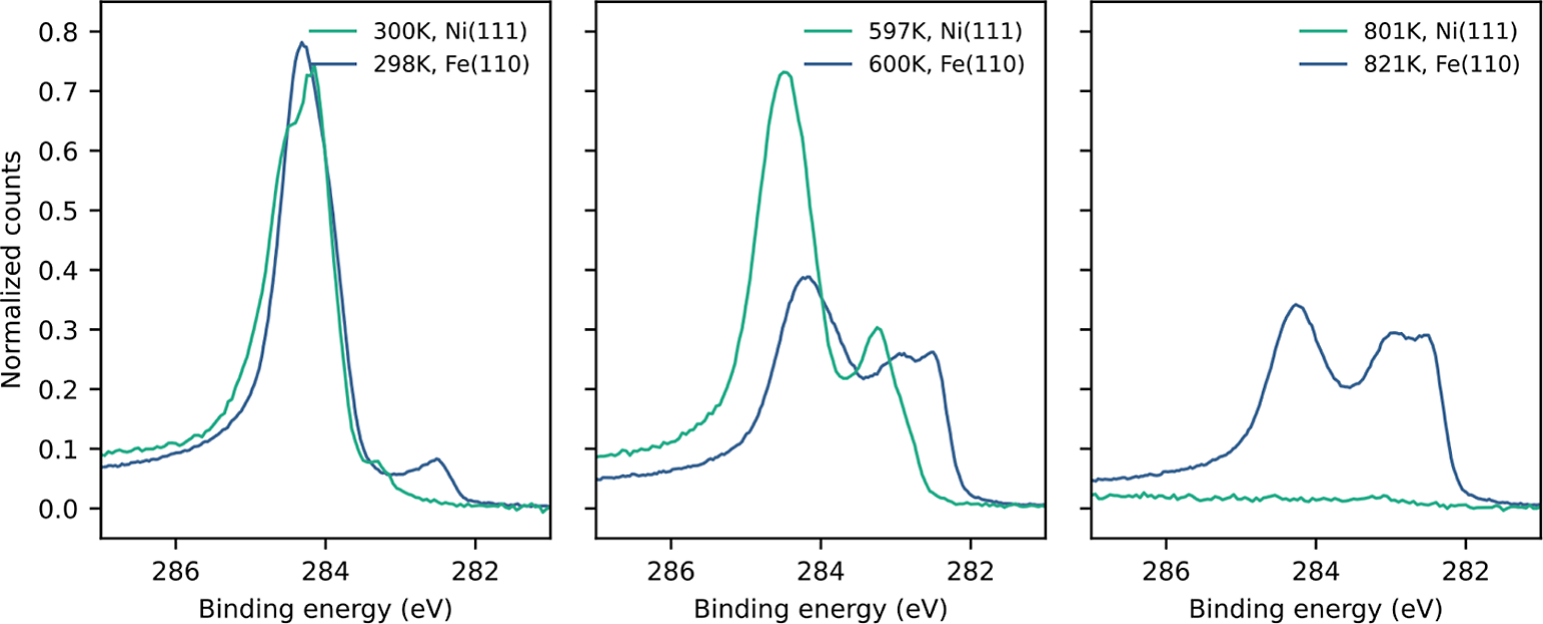
XPS spectra of 10 L naphthalene dosed on Fe(110) (in blue) and Ni(111) (in green) at three different annealing temperatures.

Figure 14 shows C 1s spectra for naphthalene on Fe(110) vs Ni(111) at selected temperatures. In the room temperature spectrum (unreacted naphthalene) on Fe(110), the main peak between 284 and 285 eV is slightly more narrow than on Ni(111). Also, whereas on nickel it is possible to distinguish C2 and C3 (with hydrogen) from C1 (without hydrogen) in the expected 4:1 ratio, this is not possible on Fe(110). Most likely, the indistinguishable peaks are caused by stronger binding on the iron surface, combined with a reaction starting at room temperature so that even at the lowest temperatures there are already more than two distinct carbon species present.

The dehydrogenation reactivity on the pristine surface is similar for the two metals, with roughly the same hydrogen desorption profile observed from both surfaces. On Fe(110) and Ni(111), the main peak at high binding energy is identifiable as graphitic carbon and coke. On nickel, this peak is favored. For iron, additional C 1s peaks at lower binding energy are clearly visible in the XPS spectra at 600 K, as shown in Figure 14 which are not seen for nickel. These lower-energy peaks indicate “chemisorbed” or atomic carbon as the main decomposition products. Thus, it seems that Fe(110) has a higher activity for carbon–carbon bond cleavage compared to nickel. However, on Fe(110), no bulk dissolution of the surface carbon takes place at high temperature (see the 800 K spectra in Figure 14). This would prevent whisker formation on catalytic particles but also means that the surface quickly becomes passivated in the case of Fe(110). While this is the case, the reactivity of the carbided Fe(110) surface only decreases very slowly from around 65% H_2_ yield left after the first cycle, as seen in the passivation experiment (see Figure 4). This could be due to the fact that the carbon is present as carbidic and chemisorbed carbon rather than C=C/graphitic carbon. The iron surface with an intermediate carbon coverage is therefore more catalytically active than what was observed for passivated Ni(111).^21^ However, the main dehydrogenation shifts to higher temperatures, indicating a increase in the reaction barrier. On Fe(110), the surface carbon does not decrease further at very high temperatures but starts to increase due to segregation from the bulk. Dissolving the carbon into the crystal bulk upon heating, as observed with Ni(111), is not possible in this case.^23^ The fitting of the C 1s spectra is more complicated than on Ni(111) due to the stronger overlap of the peaks, and the higher complexity of the spectra.

In the presence of oxygen, the naphthalene dehydrogenation temperature increases on both metals. Considering the shape of the hydrogen desorption spectrum (see Figure 9) and the total hydrogen yield (see Figure 13), the c(2 × 2) overlayer on Fe(110) does not seem to suppress the dehydrogenation to the same extent as the p(2 × 2) overlayer on Ni(111). The latter is formed between 1 and 3 L of oxygen exposure and corresponds to 0.25 ML coverage, making it easily comparable to the c(2 × 2) on Fe(110).

The complex oxidation of Fe(110), which can form multiple oxides compared to the formation of NiO on nickel, does not play a significant role in the reactivity. This can be concluded since naphthalene adsorption and decomposition are already completely inhibited on the simple oxide FeO, which is formed between 10 and 30 L.^41^ In the presence of low amounts of oxygen, “steady state” reactivity is reached, in which the surface has some carbon coverage, with additional carbon being consumed by the oxygen (see Figure 12). The dehydrogenation temperature on this surface is increased, with the first peak at 550 K (instead of 450 K), indicating a notable reduction in reaction rate. However, the total hydrogen yield from this surface is about 80% of that on the pristine surface. Since catalytic cracking of aromatic compounds is usually performed at high temperatures, a reasonable reactivity could likely be achieved.

## Conclusion

The reaction of naphthalene on Fe(110) and the effect of surface oxygen were investigated with XPS, TPD, SFG, and LEED. The direct comparison of Fe(110) and Ni(111) reveals that the initial reactivity of the clean surfaces is similar, but, as expected, Fe(110) undergoes more significant changes under varying reaction conditions, such as facile surface carbon and oxide formation. However, the iron surface exhibits a broader tolerance range in terms of reactivity, with naphthalene decomposition still occurring on a carbided and mildly oxygen covered surface. This suggests that slightly carbon covered surfaces may be optimal, as they offer greater stability than metallic iron and still exhibit a decent reactivity, implying an advantage of using iron over nickel in industrial applications where a milder, incomplete, tar reforming is desired. Additionally, the results indicate that catalytic activity could be strongly enhanced on rougher surfaces with more steps and defects, as evidenced by the substantial low temperature dehydrogenation observed on these features. The oxygen-to-carbon ratio appears to be more critical for iron, which may account for the greater variability in catalytic activity observed with iron.
